# A Case of Jaundice Revealing a Hidden Duo: Lemmel’s Syndrome and a Duplicated Gallbladder

**DOI:** 10.7759/cureus.85766

**Published:** 2025-06-11

**Authors:** Manjunath A R, Devamsh G N, Saishyam M, Nirmal Kumar M

**Affiliations:** 1 Department of Surgery, Sri Chandra Sekara Hospital, Hosur, IND; 2 Department of Gastroenterology and Hepatology, Bangalore Gastro Centre Hospitals, Bengaluru, IND; 3 Department of Gastroenterology and Hepatology, Sri Chandra Sekara Hospital, Hosur, IND; 4 Department of Surgical Gastroenterology, Sri Chandra Sekara Hospital, Hosur, IND

**Keywords:** duodenal diverticulum, gall bladder duplication, laparoscopic cholecystectomy, lemmel’s syndrome, obstructive jaundice

## Abstract

Gallbladder duplication is a rare congenital anomaly, often discovered incidentally. Its association with other biliary pathologies, such as choledocholithiasis and Lemmel's syndrome (obstructive jaundice due to periampullary duodenal diverticulum), is uncommon. This case highlights the diagnostic and therapeutic challenges in managing such a complex presentation.

A 62-year-old woman, presenting with fever, right upper quadrant pain, and deranged liver function tests, unveiled a diagnostic enigma. Initial ultrasonography suggested the presence of choledocholithiasis, though this finding was later re-evaluated. Subsequent magnetic resonance cholangiopancreatography (MRCP) identified a duplicated gallbladder, with one lobe showing signs of calculous cholecystitis, as well as an 8 mm dilation of the common bile duct (CBD) with a possible filling defect. Adding to the complexity, a large duodenal diverticulum was also identified in the MRCP. Side-viewing duodenoscopy revealed a type II major duodenal papilla. Although initial imaging suggested a filling defect in the CBD, endoscopic retrograde cholangiopancreatography (ERCP) found no stones, and the cause of the biliary obstruction remained unclear. The patient underwent laparoscopic cholecystectomy. The surgical specimen confirmed a rare duplication of the gallbladder with multiple pigment stones in one lobe and chronic cholecystitis. The dilated CBD and obstructive jaundice were attributed to Lemmel's syndrome.

This case demonstrates the rare coexistence of a gallbladder duplication and Lemmel's syndrome. Preoperative imaging is crucial for accurate diagnosis and surgical planning in patients with gallbladder anomalies. ERCP plays a vital role in both the diagnosis and treatment of Lemmel's syndrome. This case emphasizes the importance of considering rare anatomical variations and their potential impact on clinical presentation and management.

## Introduction

Gallbladder duplication, a rare congenital anomaly occurring in approximately 1 in 4,000 individuals, is attributed to the aberrant embryological development of the biliary tree [1]. Gallbladder duplication is usually asymptomatic and is often discovered incidentally during imaging studies or surgery for other reasons. However, it can be associated with an increased risk of gallstones and complications after laparoscopic cholecystectomy [2,3]. We present a unique case of a patient with gallbladder duplication harboring gallstones within one of the duplicated sacs. Notably, this individual concurrently exhibited obstructive jaundice secondary to a large periampullary duodenal diverticulum, necessitating endoscopic retrograde cholangiopancreatography (ERCP), biliary sphincterotomy, and common bile duct (CBD) stenting. To our knowledge, this constitutes the first documented instance of a patient presenting simultaneously with Lemmel’s syndrome and gallbladder duplication with cholelithiasis.

## Case presentation

**Figure 1:**
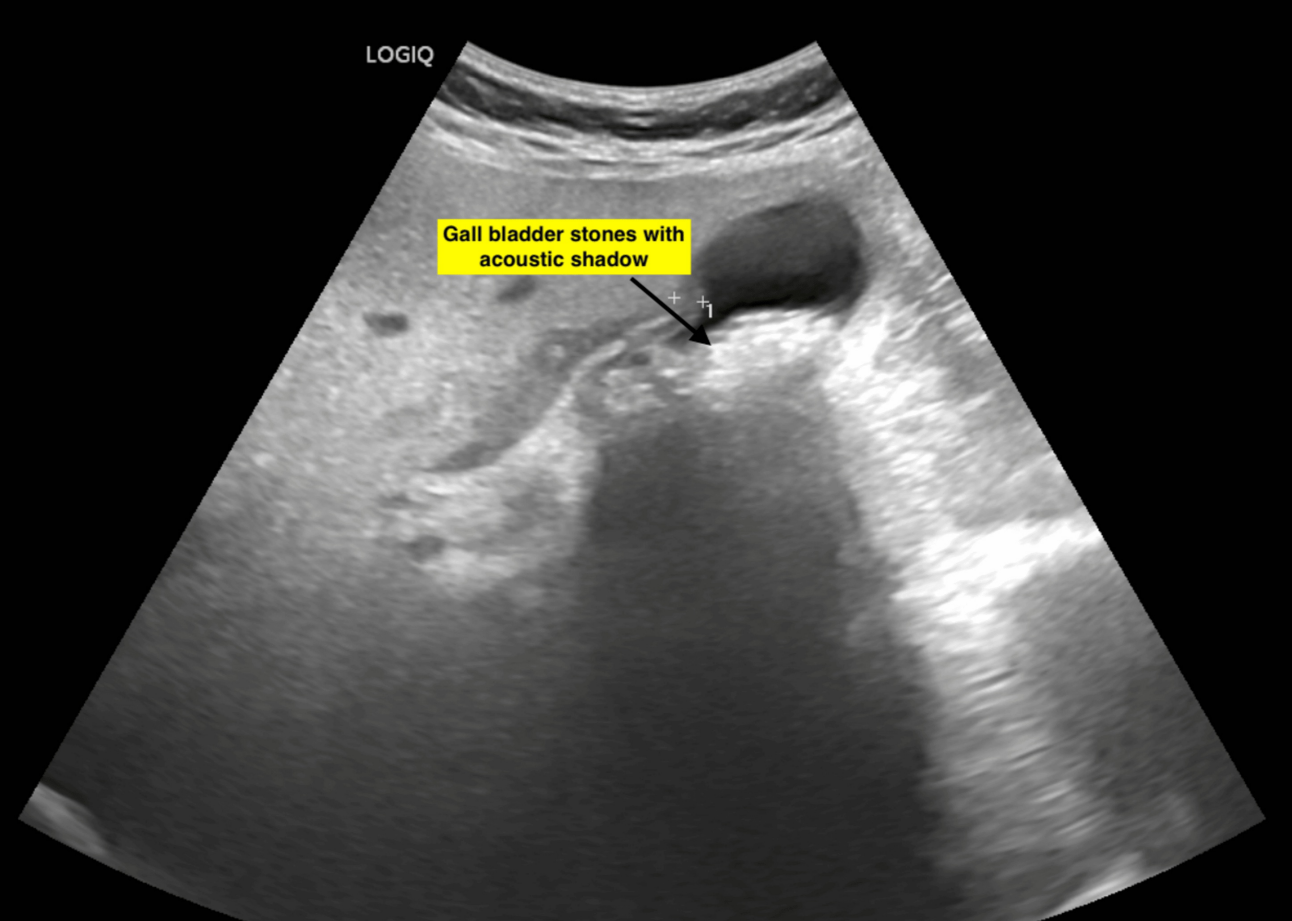
Ultrasound image showing gallbladder stones

A 62-year-old woman presented with a four-day history of fever, right upper quadrant colicky abdominal pain, and nausea. Prior medical history included diabetes mellitus and hypertension, both well-controlled with medications. Two prior episodes of biliary colic had been managed conservatively. On physical examination, right hypochondriac tenderness was noted. Icterus was absent. Ultrasound imaging revealed multiple stones within the gallbladder (Figure 1).

Laboratory investigations demonstrated elevated liver function tests: total bilirubin 2.2 mg/dL, alkaline phosphatase 138 U/L, gamma-glutamyl transferase 383 U/L, and elevated transaminases (aspartate aminotransferase 113 U/L, alanine aminotransferase 133 U/L) (Table 1). 

**Table 1:** Liver function test

Parameter	Patient value	Reference range
Total bilirubin	2.2 mg/dL	0.3-1.2 mg/dL
Direct bilirubin	1.18 mg/dL	<0.2 mg/dL
Aspartate aminotransferase (AST)	113.2 U/L	0-35 U/L
Alanine aminotransferase (ALT)	133.3 U/L	0-35 U/L
Alkaline phosphatase (ALP)	138 U/L	30-120 U/L
Gamma-glutamyl transferase (GGT)	383 U/L	5-38 U/L
Total protein	6.89 g/dL	6.6-8.3 g/dL
Albumin	3.59 g/dL	3.5-5.2 g/dL

**Figure 2:**
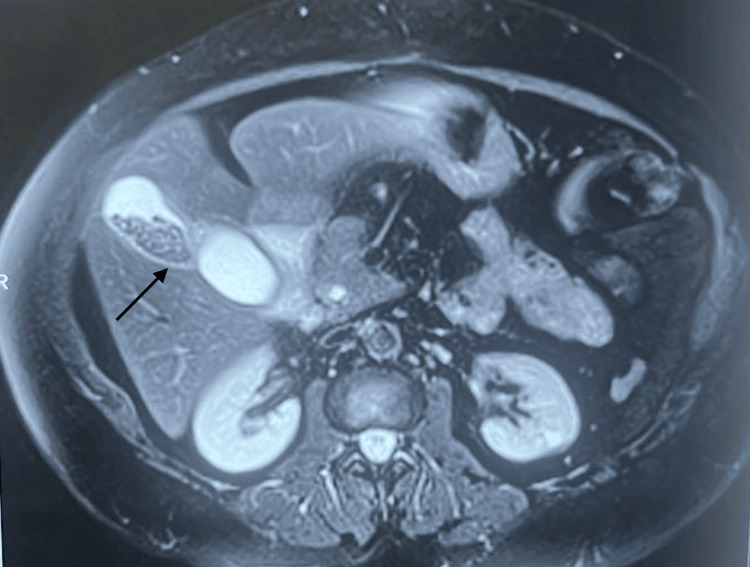
MRI image of the duplicated gallbladder with stones in one of the lobes

Magnetic resonance cholangiopancreatography (MRCP) revealed a bi-lobed duplication of the gallbladder, with one lobe exhibiting signs of calculous cholecystitis. Intrahepatic and extrahepatic biliary ducts were mildly dilated (Figure 2).

**Figure 3:**
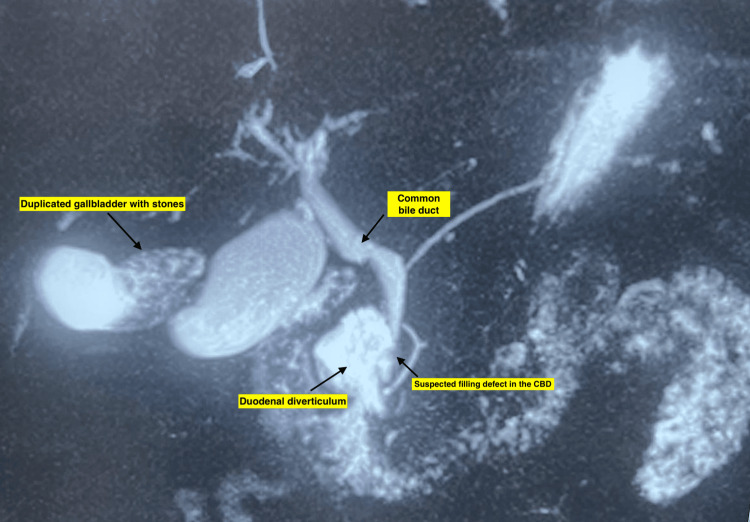
MRCP showing gallbladder duplication, common bile duct dilation, and periampullary duodenal diverticulum

The common bile duct (CBD) measured 8 mm in diameter and displayed a 3.9 x 2.7 mm filling defect, suggestive of choledocholithiasis. Notably, MRCP also identified a 4.3 x 2.1 cm duodenal diverticulum (Figure 3).

**Figure 4:**
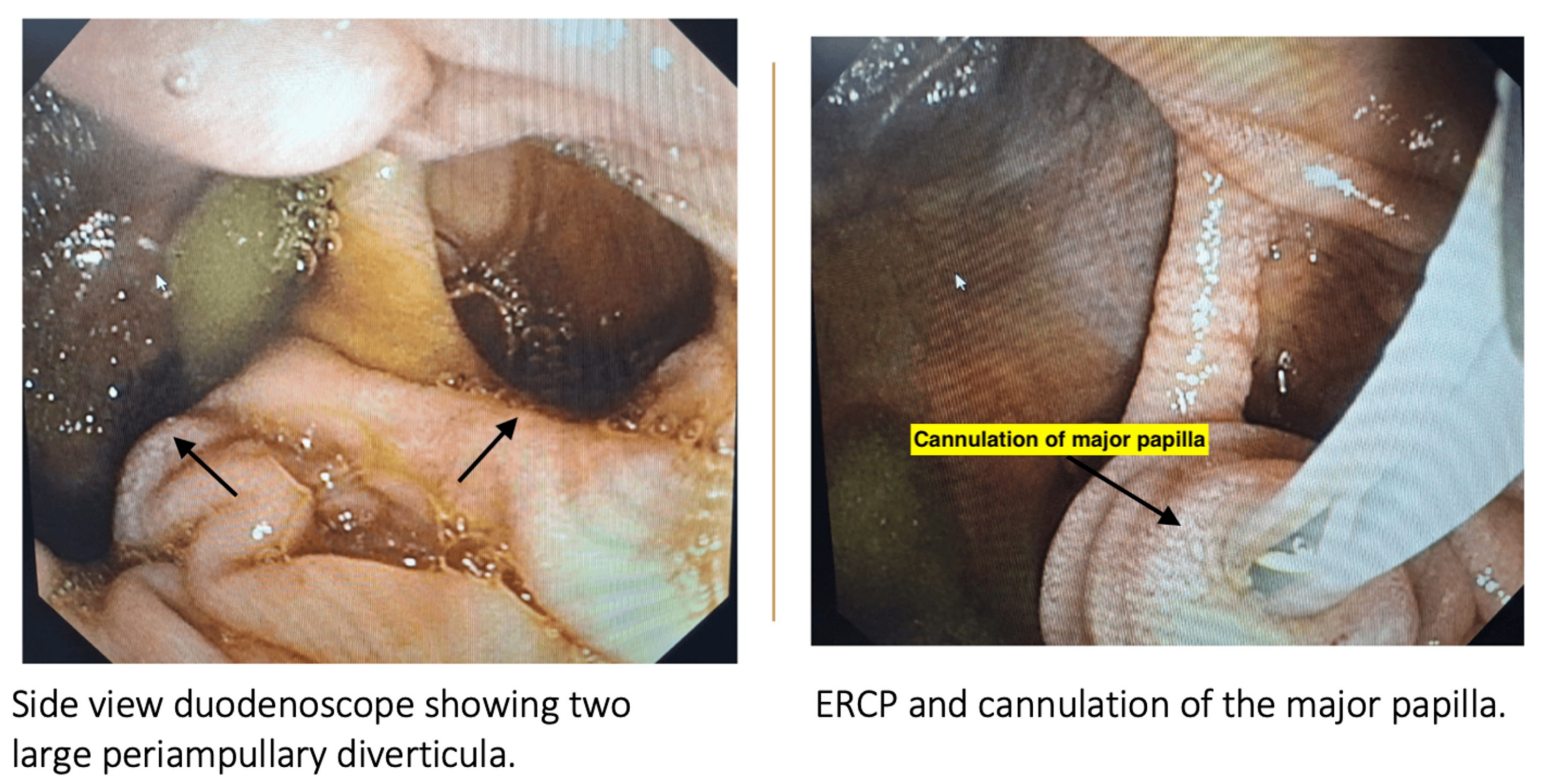
Side-viewing duodenoscopy revealing two large periampullary duodenal diverticula The image on the left-hand side shows two large duodenal diverticula near the ampullary region in the second part of the duodenum. The major papilla is located at the rim of the diverticula. ERCP was performed after cannulating the major papilla. No stones/sludge were retrieved from the common bile duct.

Side-viewing duodenoscopy confirmed the presence of not one but two large, non-bleeding, periampullary diverticula with a type II major duodenal papilla located within its margin (Figure 4).

**Figure 5:**
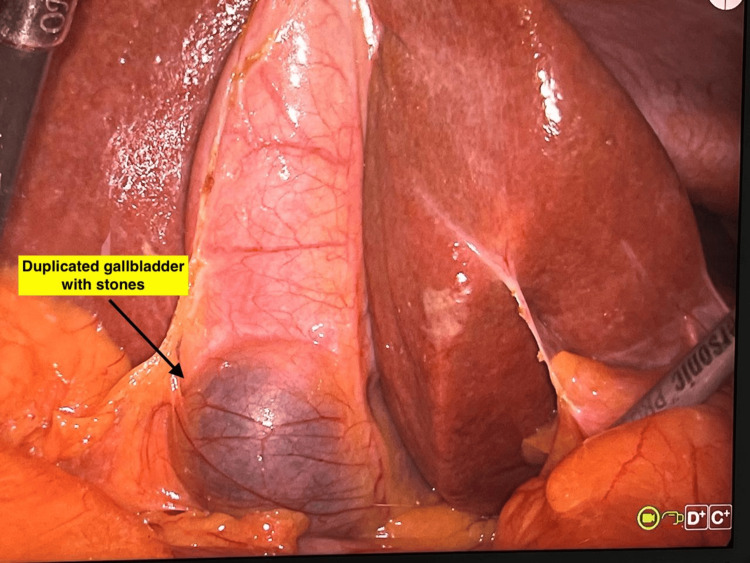
Intraoperative image of the gallbladder and undersurface of the liver

ERCP revealed a CBD of 8 mm with dilated intrahepatic biliary radicals. However, there was no stone in the CBD. Biliary sphincterotomy was performed, followed by placement of a double pigtail plastic biliary stent. Intravenous antibiotics were administered for seven days. Subsequently, the patient underwent laparoscopic cholecystectomy. Intraoperatively, mild adhesions were encountered. However, the cystic duct was clearly identified and dissected, allowing for successful cholecystectomy (Figure 5).

**Figure 6:**
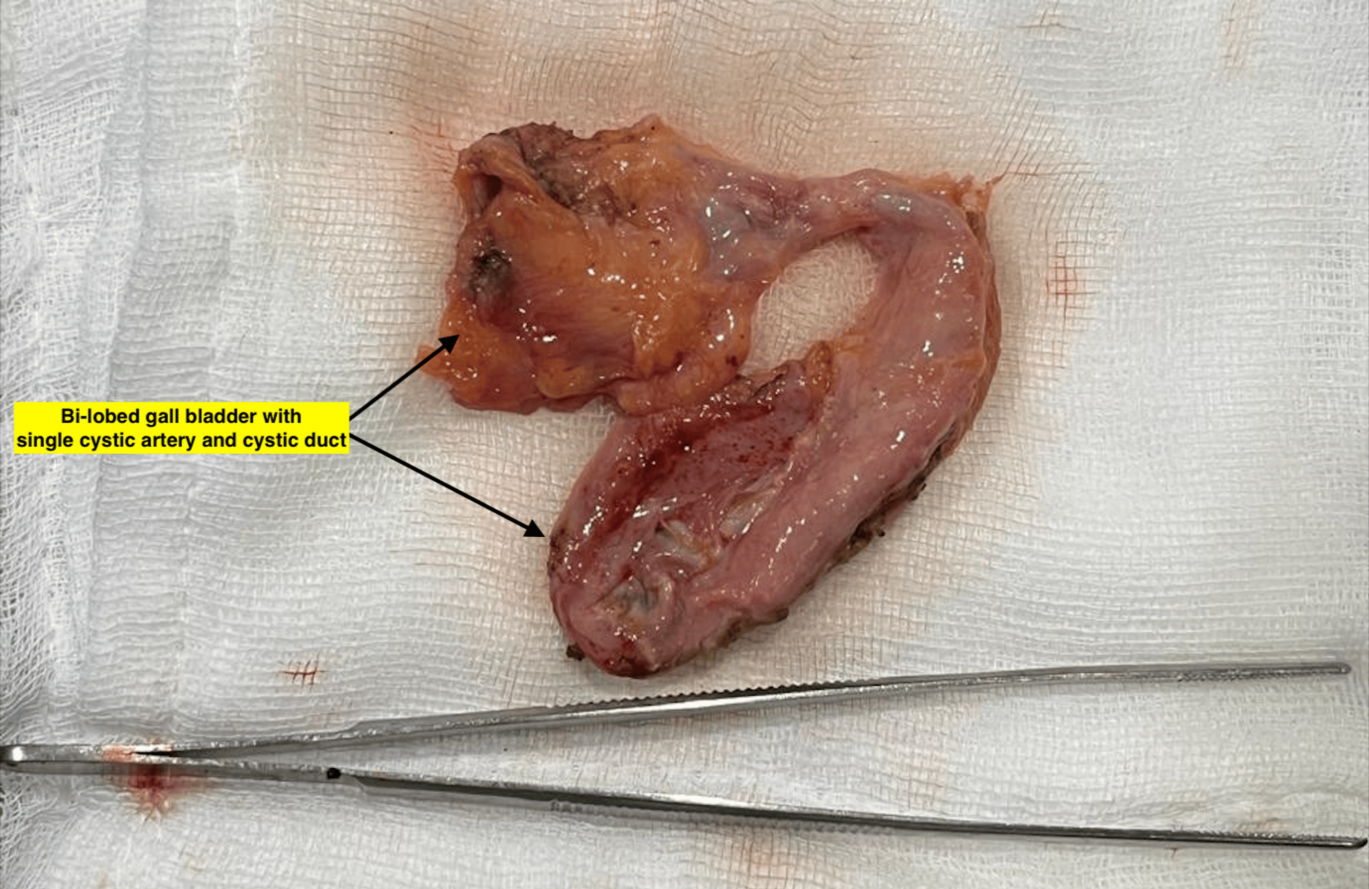
Resected specimen of the duplicated gallbladder

**Figure 7:**
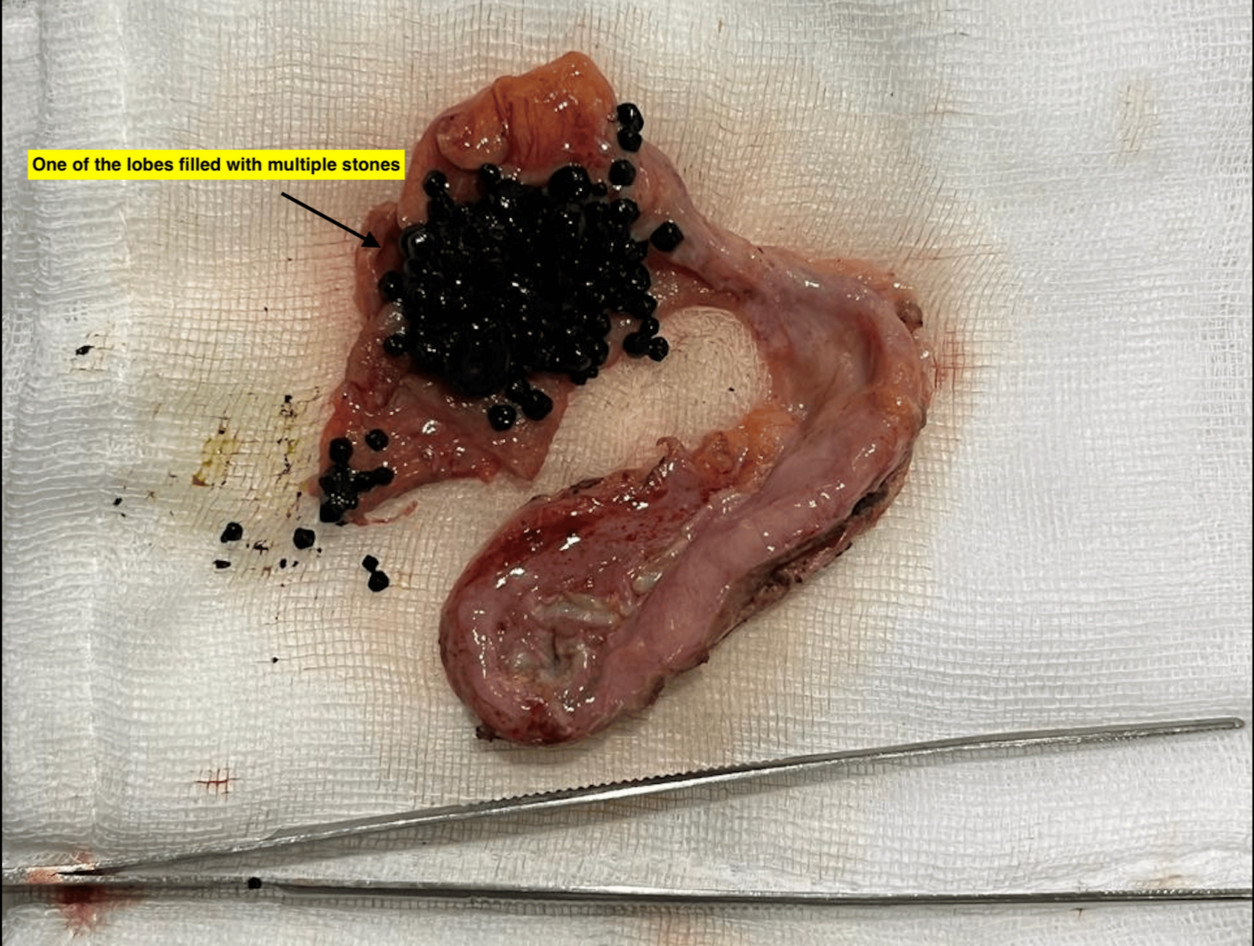
Duplicated gallbladder cut open to reveal multiple stones in one lobe

Inspection of the resected specimen revealed duplication of the gallbladder with multiple pigment stones in one of the gallbladder sacs (Figures 6, 7).

The gallbladder was bi-lobed with a single cystic duct and cystic artery. The mucosa appeared normal. Microscopic evaluation showed chronic cholecystitis. No malignancy was seen. 

## Discussion

Duplication of the gallbladder is an unusual congenital anomaly that occurs due to the splitting of the cystic primordium of the gallbladder or due to the growth of an accessory bud from the biliary primordium during embryogenesis [4]. Boyden classified them into two groups based on the presence of two separate cystic ducts [4]. Our case had vesica fellea divisa, bi-lobed with a common neck and a single cystic duct. Clinical presentation is non-specific, and the incidence of cholelithiasis appears similar to that of the general population [5]. There are no specific symptoms attributable to the duplication of the gallbladder. It goes unnoticed since most individuals remain asymptomatic. A duplicated gallbladder does not require any treatment unless patients present with biliary symptoms or develop gallbladder stones and complications. Failure to recognize gallbladder duplication and incomplete removal of the gallbladder may lead to complications like recurrent biliary colic, cholecystitis, choledocholithiasis, and biliary pancreatitis. In our case, duplication of the gallbladder was found while evaluating obstructive jaundice. Incidentally, she also had two large duodenal diverticula. Dilated CBD and obstructive jaundice were attributed to Lemmel’s syndrome after ruling out other causes. 

Lemmel's syndrome is an uncommon form of jaundice, caused by CBD compression by periampullary diverticulum. Lemmel’s syndrome may remain asymptomatic or present with diverticulitis, ulceration, bleeding, or obstructive jaundice. A recent review of the literature found that hyperbilirubinemia is the most commonly seen abnormality, followed by elevated liver enzymes and CRP [6,7]. The diagnosis of Lemmel’s syndrome can be made after demonstrating a periampullary diverticulum using CT or MRCP and ruling out other causes of obstructive jaundice. ERCP may be considered the gold standard for diagnosis [8]. ERCP is also therapeutic since biliary sphincterotomy and CBD stenting relieve obstruction in most patients. Debris in the diverticulum can also be cleared by water irrigation/lavage in patients with diverticulitis. In cases with difficult cannulation, endoscopic ultrasound (EUS)-guided or rendezvous techniques may be utilized as an alternative to biliary drainage. 

Diverticulum resection, duodenectomy, and Roux-en-Y hepaticojejunostomy with or without duodenojejunostomy have also been reported but should only be limited to symptomatic cases after failure of endoscopic treatments.

## Conclusions

Laparoscopic cholecystectomy remains the treatment of choice for a duplicated gallbladder in symptomatic patients. Preoperative evaluation of the biliary anatomy is crucial in preventing inadvertent surgical complications. Gallbladder duplication may be associated with anatomical variations in the cystic duct, hepatic arteries, and duodenal wall. Our case is unique in its presentation due to the coexistence of Lemmel’s syndrome and gallbladder duplication in the same patient.
